# Tick-Borne Encephalitis Virus: A Structural View

**DOI:** 10.3390/v10070350

**Published:** 2018-06-28

**Authors:** Lauri I. A. Pulkkinen, Sarah J. Butcher, Maria Anastasina

**Affiliations:** 1 HiLIFE—Institute of Biotechnology, University of Helsinki, 00790 Helsinki, Finland; lauri.ia.pulkkinen@helsinki.fi (L.I.A.P.); sarah.butcher@helsinki.fi (S.J.B.); 2 Faculty of Biological and Environmental Sciences, University of Helsinki, 00790 Helsinki, Finland

**Keywords:** tick-borne encephalitis virus, TBEV, flavivirus structure, envelope protein, M protein, E protein, prM, nucleocapsid, virus assembly, maturation

## Abstract

Tick-borne encephalitis virus (TBEV) is a growing health concern. It causes a severe disease that can lead to permanent neurological complications or death and the incidence of TBEV infections is constantly rising. Our understanding of TBEV’s structure lags behind that of other flaviviruses, but has advanced recently with the publication of a high-resolution structure of the TBEV virion. The gaps in our knowledge include: aspects of receptor binding, replication and virus assembly. Furthermore, TBEV has mostly been studied in mammalian systems, even though the virus’ interaction with its tick hosts is a central part of its life cycle. Elucidating these aspects of TBEV biology are crucial for the development of TBEV antivirals, as well as the improvement of diagnostics. In this review, we summarise the current structural knowledge on TBEV, bringing attention to the current gaps in our understanding, and propose further research that is needed to truly understand the structural-functional relationship of the virus and its hosts.

## 1. Introduction

Tick-borne encephalitis virus (TBEV) is a major tick-borne viral pathogen of humans. Most TBEV infections are asymptomatic, but the symptomatic cases typically have neurological manifestations, such as meningitis, encephalitis, and meningoencephalitis and, together, are referred to as tick-borne encephalitis (TBE) [1,2]. TBE is a severe disease that often results in life-long neurological complications and can lead to death [1,2]. The morbidity and mortality of TBE varies depending on the viral subtype, these are the European, the Siberian, and the Far-Eastern (TBEV-Eu, TBEV-Sib, and TBEV-FE, respectively) [1,2,3,4]. TBEV-Eu is associated with neurological sequelae in up to 10% of patients, with a 0.5–2% mortality rate, and TBEV-Sib patients are prone to develop prolonged infections with a 2–3% mortality rate, whereas TBEV-FE is associated with high rates of neurological sequelae, and up to 40% of cases are fatal [1,2]. Interestingly, the amino acid sequence variation in the polyprotein is low: up to 2.2% within and up to 5.6% between subtypes [5]. Thus, the determinants of virulence could be limited to a handful of amino acid residues in the viral proteins and/or to variable non-coding regions in the viral genome, but have not been investigated in detail [6,7]. Infection with any subtype is serious, but TBEV-FE infection is the most severe. 

TBEV is endemic to Northern Eurasia and it has been estimated that there are at least 10,000 clinical cases annually, with probable underreporting [2,8,9]. The virus is usually transmitted by ticks of the Ixodideae family, but TBEV infections can also occur via the consumption of unpasteurized contaminated dairy products [1,10,11]. Despite the availability of efficient vaccines for disease prevention, the incidence of TBE is on the rise as vaccine coverage is insufficient for many risk groups [2,12,13]. Another significant factor behind the TBE rise is global climate change, increasing the ticks’ abundance and expanding their habitats [14,15]. It is, therefore, likely that we will observe further emergence of TBEV in the upcoming decades, which calls for the development of specific antivirals for TBEV, to complement the palliative care now available [1,2].

The three TBEV subtypes are members of the genus *Flavivirus* in the family *Flaviviridae* along with other important human pathogens, such as Zika virus (ZIKV), dengue virus (DENV), West Nile virus (WNV), and Japanese encephalitis virus (JEV) [3,4,16,17]. The latter are transmitted by mosquitoes and have been extensively studied due to their significant health care threat. Tick-borne flaviviral pathogens, such as TBEV, Omsk haemorrhagic fever virus (OHFV), Powassan virus, and the emerging Alkhurma virus, have received significantly less attention compared to their mosquito-borne counterparts. Even though TBEV has been studied more than the other tick-borne flaviviruses, many of its characteristics are poorly understood. In particular, our understanding of structural details of TBEV infection is mostly based on extrapolations from the better-characterised mosquito-borne species.

The field has advanced recently with the publication of a high-resolution structure of TBEV virion alone and in complex with a neutralizing antibody, but our understanding of the details of TBEV structure and function still needs improvement [18]. In this review, we summarize current structural knowledge on TBEV, and highlight further avenues for research. 

## 2. The Structure of TBEV Particles

**Figure 1:**
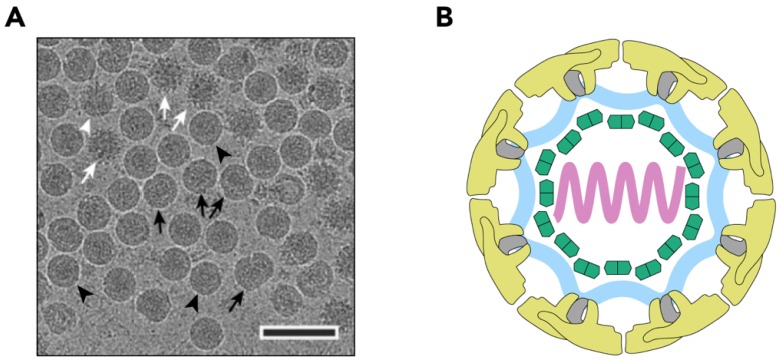

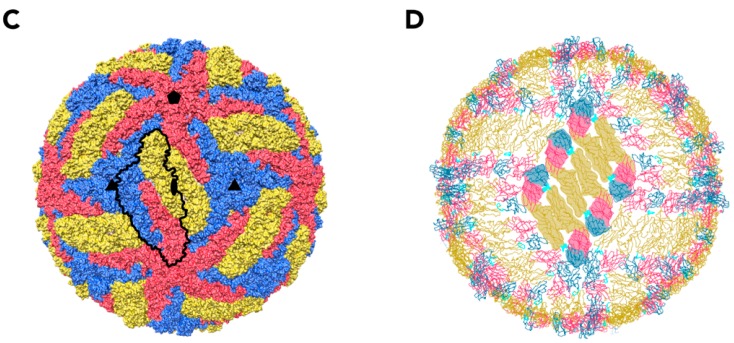
Structure of the TBEV virion. (**A**) Electron cryo-micrograph of TBEV particles purified from infected cells. Smooth mature particles (black arrowheads) are presented together with immature (white arrows), partially mature (white arrowhead), and damaged (black arrows) particles. The scale bar is 100 nm. The image is courtesy of Dr. T. Füzik et al. [14] and is reproduced under a Creative Commons Attribution 4.0 International License. (**B**) Schematic representation of the TBEV virion. Viral genome (lilac) is encapsulated by multiple copies of the C protein (green). The nucleocapsid is surrounded by a lipid membrane (light blue), in which E and M proteins (yellow and grey, respectively) are embedded; (**C**) Surface representation of the TBEV virion (wwPDB: 5O6A). An icosahedral asymmetric unit is outlined in black. The three E proteins within each asymmetric unit are shown in blue, red, and yellow. Symmetry axes are indicated by the black pentagon (five-fold), the triangles (three-fold), and the ellipse (two-fold); (**D**) Three E-M-M-E heterotetramers on the TBEV surface. Three domains of E are highlighted in red (I), yellow (II), and blue (III), and the fusion loop is highlighted in turquoise. E protein domain IV and M protein are not visible on the virion surface.

**Figure 2:**
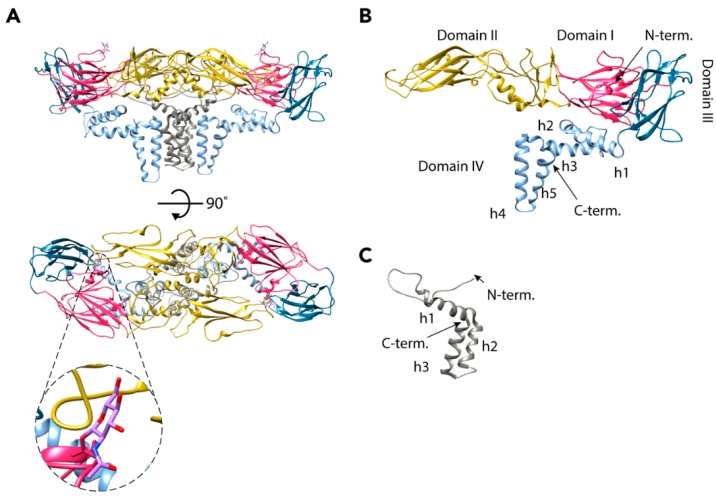
Ribbon representation of the E and M proteins as they are found in the TBEV virion. (**A**) Heterotetramer of two E and two M proteins. E proteins are coloured according to domain: red (I), yellow (II), blue (III), and light blue (IV), and M proteins are shown in grey. Zoom-in caption shows the stick representation of an Asn154 glycosylation site (pink) with an *N*-acetylglucosamine attached (violet). (**B**) The structure of the E protein monomer coloured according to domains. The five helices of transmembrane domain IV are indicated. (**C**) The structure of the M protein monomer. The peripheral membrane helix and two transmembrane helices are indicated.

TBEV has a ~11 kilobase-long positive-strand RNA (+RNA) genome that encodes a single polyprotein (UniProt: Q01299, P14336, and P07720) that is processed co- and post-transcriptionally into three structural proteins (SP) and seven non-structural proteins (nSP) [19]. Flaviviruses undergo maturation during their production, and infected cells produce at least three types of particles: immature non-infectious particles, partially-mature, and mature infectious particles (Figure 1A) [18,19,20]. The mature TBEV particles are smooth and have a diameter of 50 nm like other flaviviruses [18,21,22,23,24,25]. The virion consists of a nucleocapsid (NC) surrounded by a membrane composed of host-derived lipids in which the viral envelope (E) and membrane (M) proteins are embedded (Figure 1B) [18]. The transmembrane domains of the E and M proteins distort the lipid envelope making it slightly angular [18]. This is a common flavivirus characteristic [18,21,22,23,24,25,26]. The NC is made up of multiple copies of the capsid protein (C) and a single copy of the genome [19]. Just as with the icosahedrally-symmetric cryo-EM reconstructions of other flaviviruses, the TBEV NC is not resolved as it does not follow this symmetry, but the E and M protein are seen to ‘coat’ the lipid bilayer in an organised fashion [18,21,22,23,24,25,27]. They form heterodimers and three E-M dimers constitute the asymmetric unit of the icosahedrally-symmetric TBEV virion [18,21,22,23,24,25] (Figure 1C). The main building block of the virion is an E-M-M-E heterotetramer that is formed by head-to-tail dimerization of two E-M heterodimers (Figure 1D and Figure 2A) [18].

### 2.1. Envelope Proteins

The E glycoprotein (496 residues) is the major component of the mature TBEV particle and the X-ray structure of its ectodomains was the first flavivirus envelope protein structure solved. E consists of four domains, which are all visible in the cryo-EM reconstruction (Figure 2B) [18,28]. The N-terminal domain I forms a β-barrel structure that is central to the protein [18,28]. Domain II is elongated and consists of two areas of β-strands connected by loops and two short helices. It is the site of the dimerization interface, with a buried surface area of 14.9 nm^2^ at the interface [18,28]. Additionally, it contains the only glycosylation site of the mature virus (Asn154), which has a role in egress from mammalian cells, as well as neurovirulence (Figure 2A) [29,30]. In the cryo-EM reconstruction, a density corresponding to *N*-acetyl-d-glucosamine was observed attached to this residue [18]. At its tip, domain II also contains the highly-conserved fusion loop that is responsible for the fusion of the viral and host membranes in the final stages of TBEV entry (Figure 2A) [18,28,31]. The hydrophobic fusion loop (residues 100–109) is hidden from the aqueous environment in the hinge region between domains I and III of the other E protein in the dimer, as well as by the carbohydrate moiety of residue 154 [18,28]. Domain III of the E protein has an immunoglobulin-like fold [18,23,24,25,28,32,33,34,35]. This domain has been proposed to function in the binding to host receptors, but no residues directly responsible for entry have been identified [28,36]. Domain IV includes a stem region of three peripheral membrane helices (h1–h3) and a transmembrane region made up of two helices (h4 and h5) [18]. As the X-ray structure was of a cleaved ectodomain of E, domain IV was missing [28].

The M protein is made up of 75 residues and is therefore much smaller than the E protein. Correspondingly, it has a minor role compared to E in the mature particle, [18]. The M protein has one peripheral membrane helix (h1), two transmembrane helices (h2 and h3), and an N-terminal loop region that interacts with both E proteins in an E-M-M-E heterotetramer (Figure 2C) [18]. M is completely buried in the E-E interface, and presumably works as a ‘cement’ protein, strengthening the interaction of the E proteins [18,23]. It probably also prevents the E proteins from moving into the fusogenic conformation before the virus encounters the low-pH environment of the endosome [18,23]. The M protein is a remnant of its precursor prM (162 residues) that has a major role in the maturation of the TBEV particles (UniProt: Q01299, P14336, and P07720).

### 2.2. Nucleocapsid

**Figure 3:**
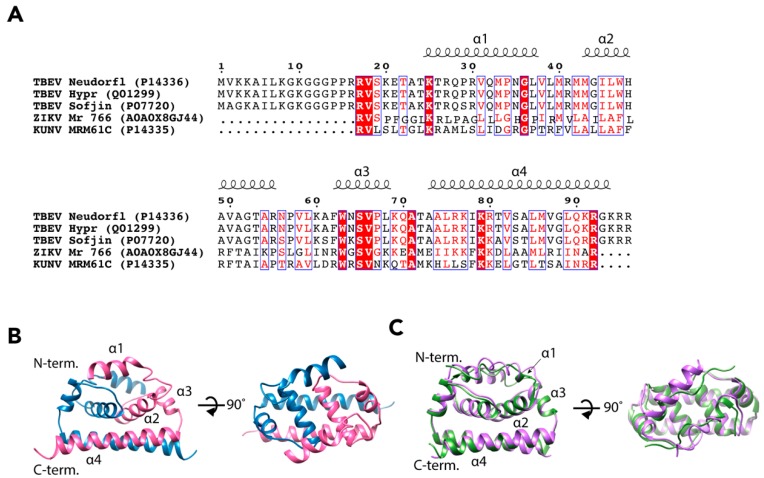
Comparison of the sequence and tertiary structure of flavivirus C proteins. (**A**) Sequence alignment of C proteins from TBEV, ZIKV and KUNV. UniProt accession numbers are shown in brackets. The alignment has been done using the Espript 3.0 web server (http://espript.ibcp.fr, [45]). White characters in red boxes highlight identical residues, red characters in blue boxes indicate residues with equivalent physico-chemical properties. Residues forming α-helices (based on the KUNV C structure) are indicated above the sequences. (**B**) Ribbon representation of a TBEV C dimer homology model (residues 24–96) built using the I-TASSER web server (C-score = −0.77) with the ZIKV C structure (wwPDB: 5YGH) as a template. The predicted positions of the α-helices are indicated. (**C**) Comparison of C-protein structures from ZIKV (green, wwPDB ID: 5YGH) and KUNV (violet, wwPDB ID: 1SFK). Positions of α-helices are indicated.

The flavivirus NCs do not follow the icosahedral symmetry of the E and M proteins so the signal is averaged out in the reconstruction process [27]. Therefore, the structure of the TBEV NC has not yet been determined [18,21,22,23,24,25,26]. It has been estimated that the molar ratio of E to C in a mature TBEV particle is close to 1:3, which would mean some 540 copies of C per virion [37]. The properties of the C protein have been investigated more thoroughly than the complete NC, and the structure of the C protein has been solved for three flaviviruses, DENV, ZIKV, and a variant of WNV, Kunjin virus (KUNV) [38,39,40]. These proteins share the same fold despite low sequence identity (Figure 3A) [38,39,40,41]. Using the ZIKV C protein (wwPDB: 5YGH) as a template, we generated a homology model of TBEV C using the I-TASSER server (https://zhanglab.ccmb.med.umich.edu/I-TASSER/), which predicted a similar overall fold with a reliable confidence score (C-score = −0.77) (Figure 3B,C) [42,43,44]. 

The C protein of TBEV consists of 96 amino acid residues (UniProt: Q01299, P14336, and P07720) and it is most likely organized into four α-helices, α_1_–α_4_ (Figure 3B,C) [38,39,40]. The C protein forms antiparallel dimers with dimerization occurring between the corresponding α_2_ and α_4_ helices of the two subunits [38,39,40]. In each monomer, the helices α_1_–α_3_ are arranged in a bundle, and the two bundles of the dimer form a hydrophobic surface that is believed to interact with host membranes [38,39,40]. In KUNV and DENV the α_1_ helices differ in orientation to each other. In contrast, the N-terminus of ZIKV C is an extended loop resulting in a much shorter α_1_ [38,39,40]. The two α_4_ helices of the dimer form a surface that is rich in basic amino acids [38,39,40]. This is most likely the RNA-binding domain of the dimer, and it is believed that RNA-C binding occurs via non-specific electrostatic interactions [38,39,40]. When crystallized, the C protein dimers were arranged in oligomeric structures: dimers of dimers in KUNV, and trimers of dimers in ZIKV [39,40]. In both cases, the authors observed channels in the middle of oligomers with RNA-binding α_4_ helices facing towards the channel interior. Therefore, they proposed that the formation of C oligomers can facilitate RNA packaging into the NC [39,40]. However, as the environment of the protein crystal is different to the complex milieu of the cell or virion, it may be that the crystal packing is not biologically relevant. Additionally, no oligomerisation of the DENV C protein dimers was observed, which may be a result of a different method of structure determination (nuclear magnetic spectroscopy versus X-ray crystallography) [38]. Alternatively, the solution structure of the DENV C dimer may reflect a different functional state than RNA packing, as the C protein has other roles during flavivirus infection [46,47,48,49].

## 3. Life Cycle

**Figure 4:**
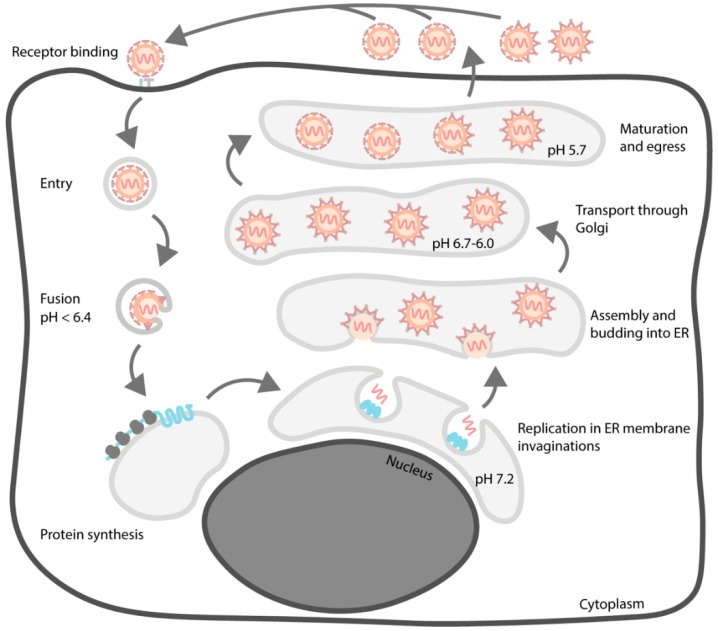
An overview of the TBEV life cycle. The virion interacts with a receptor on the cell surface and enters the cell via the endocytic pathway. The low pH in the late endosome triggers fusion of viral and endosomal membranes which leads to virion uncoating. Viral proteins are synthesized by the ribosomes of the rough endoplasmic reticulum (ER). Genome replication occurs in virus-induced invaginations of the endoplasmic reticulum (ER) and newly synthesized genomes are captured by C protein on the cytoplasmic side of the ER. The nucleocapsid complex acquires the structural proteins E and M and a lipid envelope by budding into the ER lumen through the membrane. The spiky immature particles are transported through the Golgi network and maturate in the acidic *trans*-Golgi environment after a conformational change in prM and its subsequent processing by furin. The smooth mature particles egress from the infected cell along with partially mature and immature particles. The mature and partially mature particles can start a new infection cycle but the fully immature particles are incapable of fusion and, therefore, are non-infectious.

As is the case for other flaviviruses, the assembly of TBEV particles is complex and involves multiple maturation steps [19]. Some aspects of the process, like virion maturation, are structurally quite well characterised for many mosquito-borne flaviviruses but few data are available for TBEV [50,51,52,53,54,55,56,57]. Especially, the early events of particle production remain to be elucidated. In addition, the TBEV life cycle has been mostly studied in mammalian cells, even though the tick is a central part of the biology of the virus. An overview of the TBEV life cycle is presented in Figure 4.

### 3.1. Entry

The entry process of flaviviruses occurs mainly via receptor-mediated endocytosis, but entry via micropinocytosis is also possible [58,59,60]. There are two major receptor candidates for TBEV in mammalian cells, laminin-binding protein (LBP) and the αVβ3 integrin but no receptor candidates in tick cells have been identified so far [61,62,63]. Studies using anti-idiotypic antibodies suggested that there could be other receptor candidates of as yet unknown identity [61,64,65]. In addition, under certain conditions it is possible to fuse TBEV with liposomes, hence, lipids could also be involved in binding [66,67]. For other flaviviruses, various receptor candidates have been proposed, which could be also be relevant for TBEV (reviewed in [68]).

In addition to entry receptors, *sensu stricto*, it has been proposed that TBEV utilizes attachment factors that bind the virus on the cell surface without initiating endocytosis. The most prominent of these is heparan sulphate, a glycosaminoglycan that works as an attachment factor for multiple viruses [69,70,71,72,73,74]. Even though utilisation of heparan sulphate is a commonly seen cell culture adaptation of TBEV, it is also present in some wild-type isolates. The binding between heparan sulphate and TBEV particles occurs via the E protein, and cell culture-associated adaptations mainly manifest as mutations that increase the positive charge of the E protein [71,73,75].

The carbohydrate moiety of the E protein has been shown to be dispensable for TBEV entry in cell culture [30,76]. However, the lack of E glycosylation leads to reduced neuroinvasiveness in mice, which may indicate that interaction with a carbohydrate-binding protein has a role in TBEV attachment to neurons [30]. This is further supported by the observation that in a closely related tick-borne flavivirus, Louping ill virus, a mutation in the glycosylation site reduced neurovirulence [77]. The binding of DENV to its attachment factor, dendritic cell-specific ICAM3 grabbing nonintegrin, is mediated by the interaction of the carbohydrate recognition domain and the E protein Asn67 carbohydrate moieties of the virus [78]. Even though this residue is not glycosylated in TBEV, a similar interaction may occur using the glycosylated Asn154 instead. 

TBEV can be endocytosed and cause infection, when bound by non-neutralising quantities of antibodies [79]. This phenomenon is known as antibody-dependent enhancement (ADE) and it has been shown *in vitro* for multiple flaviviruses (reviewed in [80]) and recently on the epidemiological level for DENV [81]. ADE is mediated by the binding of the virus-antibody complexes to Fcγ receptors in the host cell surfaces but, recently, a new ADE mechanism was identified for TBEV. This mode of entry is independent of Fcγ receptors and other cell surface proteins and is proposed to be mediated by antibody-mediated exposure of the E protein fusion loop which then binds directly to host membranes [82].

**Figure 5:**
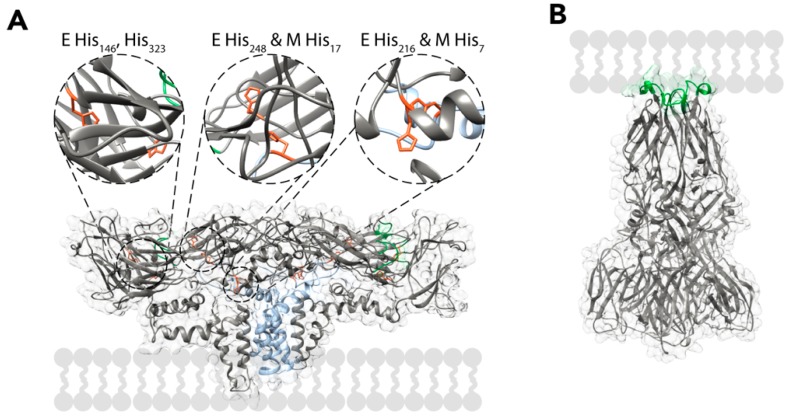
Conformational rearrangement of the E protein during fusion. (**A**) Ribbon representation of the E-M dimer at neutral pH (wwPDB: 5O6A). Zoom in captions show pH-sensing histidines in detail. The E protein is shown in grey, the M protein is shown in light blue, the fusion loop is shown in green, and the pH-sensing histidine residues are shown in orange. The lipid bilayer is shown schematically. (**B**) Post-fusion E trimer conformation (wwPDB: 1URZ). The E proteins are shown in grey and fusion loops are shown in green. The lipid membrane is schematically shown. Domain IV of the E protein and protein M are not shown.

After the TBEV particles have entered the cells, the virions are localised inside endocytic vesicles. In the endosome, the pH progressively drops, which leads to major rearrangements in the virion. Mutagenesis studies of TBEV E proteins have implicated the protonation of His323 (and possibly His146) as the main pH detection mechanism, but the cryo-EM reconstruction of the virus implies that other histidines have pH-related roles as well. From a structural perspective, it seems likely that the protonation of histidines in the E and M proteins would cause them to repel each other, destabilising the heterotetramer and exposing the fusion loops. The residues implicated for this are His216 and His248 of the E protein and His7 and His17 of the M protein (Figure 5A) [18,23,83]. The fusion loops embed into the membrane of the endosome, possibly with the help of the detachment of the peripheral membrane helices of E from the viral envelope [84,85,86]. After binding to the membrane, E proteins trimerize via the interaction of the fusion loops [84,87]. In the current model, this pre-fusion trimer undergoes a hairpin-like conformational change that brings the membranes of the endosome and the virus into close contact forming a post-fusion trimer (Figure 5B) [88,89]. The post-fusion trimers are then stabilized by interactions between domains I and II, as well as the peripheral and transmembrane helices of the different subunits [90,91,92,93]. The conformational change from a pre-fusion to a post-fusion trimer allows the fusion of the viral and endosomal membranes via a hemifusion intermediate. This leads to the release of the NC into the cytosol [89,94]. Membrane fusion is dependent on the correct lipid composition, and cholesterol strongly enhances it [66,67,87]. After the NC has entered the cytosol, it disintegrates and releases the viral RNA. The events responsible to the uncoating of the TBEV RNA have not been elucidated, but for DENV, it has been shown that the dissociation of the NC requires non-degradative ubiquitination [95].

### 3.2. Replication and Translation

**Figure 6:**
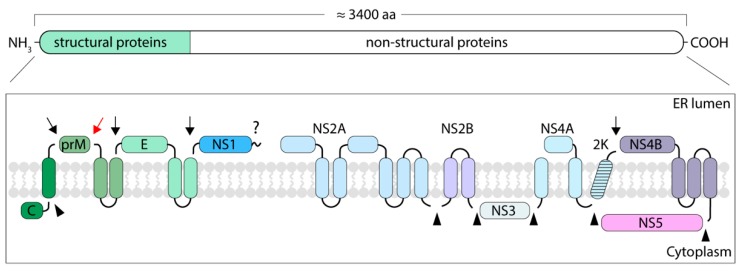
Schematic representation of the flavivirus polyprotein and its cleavage products. Structural proteins are shown in shades of green and non-structural proteins are shown in shades of blue, shades of violet, and in pink. Black arrows indicate viral serine protease cleavage sites, triangles indicate host signal peptidase cleavage sites, the question mark indicates the cleavage site of an unknown host protease, and the red arrow indicates a furin cleavage site. The ER membrane is shown in grey and the ER lumen and the cytoplasm are indicated.

In infected cells, the TBEV genome is translated at the endoplasmic reticulum (ER) as a single polyprotein. The polyprotein is cleaved by viral and host enzymes to yield SPs that form the virion and nSPs that are responsible for genome replication, polyprotein processing and modulation of cellular functions (Figure 6) [19]. TBEV SPs have been studied in detail, but the current knowledge on flavivirus nSPs mostly comes from studies of mosquito-borne species. Most of the TBEV proteins are proposed to be either integral membrane proteins or to have membrane anchors, some of which are cleaved during polyprotein processing (UniProt: Q01299, P14336, and P07720) [19].

The mature C is a soluble cytoplasmic protein as its membrane anchor is cleaved but the prM and E proteins localise in the lumen of the ER, where they remain bound to the membrane by double-helical anchors that are typical to flaviviruses [19]. The cleavage of the C-terminal membrane anchor first from C and then from prM is sequential and strictly controlled. In Murray Valley encephalitis virus, YFV, and WNV, the perturbation of the cleavage order results in excessive formation of NC-deficient particles [96,97,98,99]. For TBEV, however, the uncoupling of these events only affects particle production in tick cells [97].

The first translated nSP in flaviviruses is the NS1 protein that localises in the lumen of the ER (reviewed in [100]). It is a multi-functional protein that exists in dimeric and hexameric forms. As a dimer, NS1 has a role in replication whereas as a hexamer it is co-secreted with TBEV particles and modulates the complement system of the mammalian host. The immunomodulatory activity of NS1 results in reduced formation of membrane attack complexes and, therefore, prevents the destruction of infected cells [100]. It also reduces the inactivation of extracellular viruses by binding to the C4 component of the complement system [101]. NS2A and NS2B are both integral membrane proteins with roles in particle assembly. NS2A functions in replication and immunomodulation whereas NS2B is a co-factor for the NS3 protease [102,103,104,105,106]. The NS2B-NS3 complex has both protease and helicase activities and it is responsible for the viral enzyme-mediated cleavage of the polyprotein (reviewed in [107]). The helicase domain of NS3 has an ATPase activity that is regulated by the integral membrane protein NS4A [108]. NS4A is separated from NS4B by a signal sequence called the 2k peptide that directs NS4B to the ER membrane and is later cleaved off by the host signal peptidase. After 2k cleavage, NS4B remains integrated in the ER membrane where it performs multiple functions from replication complex formation to immunomodulation (reviewed in [109]). The flaviviral genome is replicated by the RNA-dependent RNA polymerase NS5, which has an immunomodulatory role as well (reviewed in [110,111]). In addition to the nSPs, the C proteins of flaviviruses have multiple regulatory roles during the infection, including immunomodulation and the prevention of nucleosome formation [46,47,48,49]. No TBEV nSP structures have been solved. The nSP structures available for other flaviviruses have been studied using X-ray crystallography of purified proteins, which makes it difficult to provide a full structural picture of flaviviral replication in the context of the infected cell [112,113,114].

The replication of TBEV occurs in a close contact with the ER membrane, which is extensively rearranged by NS1, NS2B, NS4A, and NS4B [100,104,115,116,117,118,119,120,121,122,123]. In tick-borne flaviviruses these rearranged ER membranes are observed in both tick and mammalian cells, but in tick cells the membrane rearrangements are less prominent. Corresponding to a slower rate of replication in the tick cells, fewer particles are also observed than in mammalian cells [117,124]. The replication of the TBEV genomes occurs via a dsRNA intermediate in ER invaginations. The invaginations have ‘necks’ that connect to the cytosol, presumably allowing nucleotides to enter and the RNA genomes to exit [19,115,117,120,121,123].

### 3.3. Assembly and Budding

The newly-synthesized viral genomes are encompassed by multiple copies of the C protein to form NCs. Based on the structures of the C proteins, it seems that the NCs are formed by electrostatic interactions between the C-terminal α_4_ helices of C and viral RNA [38,39,40]. This suggestion is corroborated by data showing C proteins of ZIKV and DENV bind various types of nucleic acids regardless of the sequence [40,125]. Furthermore, recombinant DENV C protein dimers bind double-stranded DNA of various lengths, forming capsid-like particles (CLP) [125]. Overall, the packaging of flaviviral genomes is a robust process, as the C proteins can remain functional despite large-scale deletions [41,126,127,128]. In a YFV-based reporter system, it was even noticed that assembly required either the α_4_ helix or the N-terminal basic residues, but not both [41,128]. The TBEV C protein is similar to other flaviviruses: it can remain functional despite internal deletions, it binds various nucleic acids without signal specificity, and CLPs can be produced from purified C protein and nucleic acids [126,129]. This suggests that the assembly of TBEV NCs is analogous to other flaviviruses.

As the C proteins can package RNA regardless of sequence, a spatial and temporal coupling of replication, translation, assembly, and budding has been proposed to explain how flaviviruses manage to specifically pack their genomes (reviewed in [130]). Several lines of evidence support this hypothesis: in DENV-infected cells budding into the ER lumen occurs directly opposite to or in close contact with the vesicular structures where the genome is replicated. In KUNV only actively-transcribed RNA is packaged [131,132,133]. Furthermore, many of the nSPs that localize at the sites of replication have also been implicated in particle assembly. Functional NS2A is required for the assembly of KUNV, DENV, and YFV particles, the transmembrane domains of NS2B and its binding partner are required for JEV particle formation, and NS3 has been implicated in particle assembly in YFV and KUNV [104,105,134,135,136,137,138,139]. It is also possible that even though RNA-C protein binding is sequence-independent, the specificity of RNA packaging is mediated by genomic assembly signals that target one or multiple nSPs instead of C. However, the binding of nSPs to the only candidate for a flaviviral packaging signal, CCR1, has not been studied. Furthermore, the data supporting its role in assembly is indirect [140].

The particle and NC assembly processes do not solely rely on viral proteins. In JEV-infected cells, transmembrane domains of NS2B interact with the host factor SPCS1 to secure particle production and in DENV infections, the interaction of C protein and nucleolin is essential for formation of virions [141,142]. Additionally, WNV particle production requires the presence of the host helicase DDX56 at the viral assembly sites [143,144]. However, the host factors required by TBEV particle formation are not known. In contrast, it has been recently shown that the host protein viperin prevents TBEV assembly by promoting the production of non-infectious particles containing solely C protein and a membrane [145]. The detailed characterisation of other antiviral host factors in TBEV infection is outside the scope of this article and has been reviewed elsewhere [146].

Once the NCs have been assembled, they acquire their lipid envelopes by budding into the ER lumen. The budding, however, can occur without the presence of the NC, as the production of NC-deficient subviral particles is a normal part of flavivirus infections [20]. Fusion-competent subviral particles can also be produced by recombinantly expressing prM and E in cells, which implies that the budding process is mediated by the lateral interactions of these proteins [66,147,148]. The structural details of budding have not been elucidated, but it seems that the interaction of the prM and E protein transmembrane helices is required [149]. Although budding can occur without the assembly of NCs, the events need to be coupled as flavivirus-infected cells rarely produce mainly empty particles and naked NCs are generally not observed in infected cells [19,120,121,123,124]. 

### 3.4. Particle Maturation and Egress

**Figure 7:**
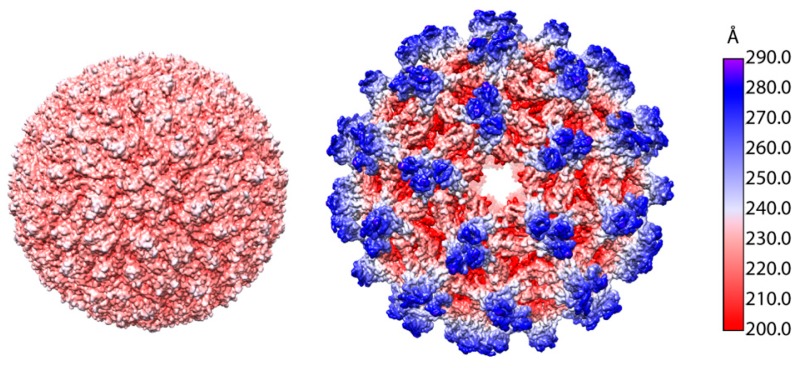
Surface view of the mature and the immature DENV particles (wwPDB: 3J27 and 4B03, respectively) coloured radially according to the key. Only the prM and E models are included, the membrane and nucleocapsid inside the immature particle is not visible, leading to apparent ‘holes’ on the five-fold axis of symmetry.

The immature flavivirus particles formed by budding through the ER differ greatly from their mature, infectious forms. Even though the immature TBEV particle has not been structurally characterized, it is presumed to be similar to the flaviviruses for which the structure of this intermediate form is available [50,52,56,57] (Figure 7). In the immature particles, the pr peptide has not been cleaved from M yet, and the particles consist of heterodimers of prM and E. The cryo-EM reconstructions of both naturally occurring and artificially induced immature flavivirus particles consistently reach lower resolution than those of mature virions. This implies flexibility that is not present in the mature virions [24,52,56,57]. The immature particles are larger than the mature forms, which is due to the organisation of the prM-E dimers into trimeric spikes [50,52,56,57]. In the immature particles, the pr peptides coat the fusogenic loops of the E proteins, preventing premature fusion [50,52,56,57,150]. In DENV and ZIKV, the prM is glycosylated in the area directly on top of the fusion loop (Asn69), which increases the hydrophilicity of the spike tip, presumably to prevent interaction with the ER membrane [53,57]. In TBEV the glycosylated residue is Asn25 (UniProt: Q01299, P14336, and P07720). In the DENV and ZIKV pr structures, residues 25 and 69 are close together, indicating that the glycosylation of either could have a similar effect, which suggests that pr glycosylation in TBEV has the same role as in DENV and ZIKV [53,57]. Each spike is stabilised by the interaction of the pr proteins at the tip and by the interactions between domains II and III of neighbouring E proteins. These connections, however, are not very strong, which may contribute to the lability of the immature particle [53,56]. In most reconstructions of immature flavivirus particles, the NC density remains poorly resolved, but in the immature ZIKV particle, a density corresponding to C protein was observed under the trimeric spikes, suggesting partial organisation of the NC [50,52,56,57]. Since this ordered density is not visible in the mature virion, it implies that during the maturation process the NC undergoes a conformational change [24,57].

The current model for flavivirus maturation was first established with TBEV using biochemical and molecular biology methods [151,152,153,154,155]. Later, this model was supported via structural studies of mosquito-borne flaviviruses [53,54,56]. After the immature particles form by budding into the ER, they pass through the Golgi apparatus and the *trans*-Golgi network (TGN) [19]. In the TGN, the particles are exposed to low pH, which causes a major conformational change in the (prM-E)_3_ spikes. The spiky immature particles change into smooth ‘pre-mature’ particles as the trimeric spikes dissociate, and the prM-E dimers further dimerize, forming a structure similar to the mature particle. The only difference is the presence of the pr peptide, which is still localised on top of the fusion peptide [53,54,56]. The rearrangement of the spikes begins in one or more independent nucleation centres instead of occurring simultaneously across the particle, as this would lead to steric clashes [55]. Interestingly, this conformational change is reversible in DENV and irreversible in TBEV, indicating possible differences between the maturation of mosquito-borne and tick-borne flaviviruses [54,153].

No specific pH-sensing residues have been implicated for maturation in TBEV but it is tempting to speculate that the same histidines that are needed for the conformational changes leading to membrane fusion would have a role in this process as well [18,83]. Indeed, His244 in E and His98 in prM are needed for the formation of pre-mature DENV particles and they are presumably protonated during the maturation process [156,157]. In TBEV, there are histidines in comparable positions in E and prM (His248 and His95, respectively) and it is, therefore, likely that they have similar roles in TBEV maturation (UniProt: Q01299, P14336, and P07720). Based on the structure of the mature TBEV particle, these residues may also function as pH sensors during fusion (His95 of prM is in position 7 in M after pr is cleaved) [18].

After the conformational change, the maturation is completed by the cleavage of the pr peptide from prM by the host protease, furin. In the immature particle, the prM furin cleavage site is inaccessible. After the pH-mediated conformational change, it is exposed and the pr peptide is cleaved. However, it still remains bound to the E-M-M-E heterotetramer at the acidic pH of the TGN. Therefore, it still obscures the fusion loop, preventing premature fusion with the TGN membranes. The pr peptides can only dissociate from the virion after it exits the cell via endocytosis and reaches the neutral extracellular milieu. The pr dissociation primes the virion for fusion, thus rendering it infectious [53,54,156,157].

The maturation process in flaviviruses is not always complete, which leads to the production of immature and partially mature particles by the infected cells. The fully immature particles are non-infectious because they are incapable of fusion, but the partially mature particles can infect new cells [152,153]. The partially mature particles are structurally and antigenically heterogenous. Their production has been suggested to act as an immune evasion strategy (reviewed in [158]) and as a way to increase the range of tissue tropisms (reviewed in [159]).

For TBEV, the process of maturation and egress has mainly been studied in mammalian cells, but limited evidence shows there may be differences between the mammalian and tick systems. The glycosylation of E protein is required for egress in mammalian, but not in tick, cells [29,30,76]. Additionally, in tick cells blocking the transport from the ER to the Golgi apparatus did not reduce virus production [30]. In some electron microscopy studies, it has been reported that the entire process of flavivirus assembly and maturation in tick and mosquito cells differs from mammalian cells. In these reports, pre-formed NCs have been observed associated with various host cell structures like phagosomes to acquire their membranes via budding through the plasma membrane [117,160]. These findings have not, however, been confirmed with other approaches.

## 4. Future Perspectives

It is clear that many aspects of TBEV biology remain unknown, even though there have been considerable advances in flavivirus research in recent years. The TBEV life cycle is complex, and we can better understand viral assembly, maturation, and entry by the structural characterisation of the different intermediate forms of the virus particles. The TBEV NC is a tempting target for study: it is difficult to approach but can provide important knowledge about uncoating and assembly, the two most enigmatic stages of the flavivirus life cycle. Understanding NC assembly may help to answer the question of how TBEV specifically packages its genome despite the apparent sequence-agnosticism of the C protein.

The multi-functional nSPs of TBEV are critical for infection and could be determinants of virulence, which makes them important targets for structural and functional studies. They would provide essential information about TBEV genome replication, particle assembly, virus-host interactions, and immune evasion. We can decipher the nSPs’ mechanistic roles in infection by combining structural and in situ approaches. These studies could also yield novel drug targets, as exemplified by the current development of nucleoside analogues that block the function of the NS5 protein and reduce TBEV neurovirulence in vivo [161,162].

The virulence factors responsible for the different TBEV subtype pathologies have not yet been comprehensibly examined. Tissue tropism could explain the clinical differences between the TBEV subtypes. Therefore, this variation may be investigated by studying TBEV entry into different cell types. A number of residues that vary across TBEV subtypes have been localised to the E protein, which is responsible for receptor interaction [5]. Hence, by identifying the TBEV receptor(s) and studying interactions with the virus we may better explain TBEV pathogenicity.

Finally, despite its obvious importance to TBEV biology, the virus has been poorly studied in ticks. Although research in mammalian systems is warranted, it needs to be combined with investigations in tick systems for a more complete understanding of TBEV biology and emergence.
